# High-contrast three-dimensional imaging of the Arabidopsis leaf enables the analysis of cell dimensions in the epidermis and mesophyll

**DOI:** 10.1186/1746-4811-6-17

**Published:** 2010-07-02

**Authors:** Nathalie Wuyts, Jean-Christophe Palauqui, Geneviève Conejero, Jean-Luc Verdeil, Christine Granier, Catherine Massonnet

**Affiliations:** 1 Laboratoire d'Ecophysiologie des Plantes sous Stress Environnementaux, INRA-SupAgro, 2 Place Viala, 34060 Montpellier, France; 2 INRA, Centre de Versailles, Institut Jean-Pierre Bourgin, Route de Saint-Cyr, 78026 Versailles, France; 3 Plate-forme d'Histocytologie et d'Imagerie Cellulaire Végétale, Biochimie et Physiologie Moléculaire des Plantes-Développement et Amélioration des Plantes, INRA-CNRS-CIRAD, TA96/02 Avenue Agropolis, 34398 Montpellier, France

## Abstract

**Background:**

Despite the wide spread application of confocal and multiphoton laser scanning microscopy in plant biology, leaf phenotype assessment still relies on two-dimensional imaging with a limited appreciation of the cells' structural context and an inherent inaccuracy of cell measurements. Here, a successful procedure for the three-dimensional imaging and analysis of plant leaves is presented.

**Results:**

The procedure was developed based on a range of developmental stages, from leaf initiation to senescence, of soil-grown *Arabidopsis thaliana *(L.) Heynh. Rigorous clearing of tissues, made possible by enhanced leaf permeability to clearing agents, allowed the optical sectioning of the entire leaf thickness by both confocal and multiphoton microscopy. The superior image quality, in resolution and contrast, obtained by the latter technique enabled the three-dimensional visualisation of leaf morphology at the individual cell level, cell segmentation and the construction of structural models. Image analysis macros were developed to measure leaf thickness and tissue proportions, as well as to determine for the epidermis and all layers of mesophyll tissue, cell density, volume, length and width. For mesophyll tissue, the proportion of intercellular spaces and the surface areas of cells were also estimated. The performance of the procedure was demonstrated for the expanding 6^th ^leaf of the Arabidopsis rosette. Furthermore, it was proven to be effective for leaves of another dicotyledon, apple (*Malus domestica *Borkh.), which has a very different cellular organisation.

**Conclusions:**

The pipeline for the three-dimensional imaging and analysis of plant leaves provides the means to include variables on internal tissues in leaf growth studies and the assessment of leaf phenotypes. It also allows the visualisation and quantification of alterations in leaf structure alongside changes in leaf functioning observed under environmental constraints. Data obtained using this procedure can further be integrated in leaf development and functioning models.

## Background

Eighteen years have passed since the need for a comprehensive three-dimensional anatomical description of the cellular structure of an *Arabidopsis thaliana *(L.) Heynh. leaf was first formulated [1]. The detailed characterisation of the cellular structure of the wild-type leaf would provide the factual basis for the identification of even the most subtle phenotypes of mutants and aid in the unravelling of growth mechanisms. A large number of Arabidopsis genotypes dramatically affected in leaf form or dimensions and in cell numbers or dimensions have been identified [2]; however, potentially many other genotypes have informative leaf phenotypes which are currently not detected. Besides genetic factors, environmental conditions affect leaf expansion rates and duration [3] and bring about morphological changes in leaf tissues which relate directly to leaf functioning in photosynthesis and transpiration [4-9]. Models of leaf size control integrate data on quantitative growth variables at the plant, organ and cell level, but for the latter, only epidermal cells or the sub-epidermal layer of palisade mesophyll cells are taken into account, not the whole leaf thickness [10-12]. A three-dimensional structural model of the developing leaf does not exist as yet, because the essential quantitative parameters of internal leaf tissues are missing.

The routine methods for leaf phenotype assessment at a cellular level use brightfield or differential interference contrast microscopy. Cell length measurements are performed on transverse sections obtained by classical histological sectioning with the inherent spatial inaccuracy [1,13], while cell area or width is determined using epidermal peels or paradermal views of cleared leaves with measurements limited to the epidermis and one sub-epidermal layer of mesophyll cells [10-17]. Cell separation is another option for area measurement of mesophyll cells, but it implies the loss of any information on cellular organisation [1,18,19]. Important progress in the understanding of the processes of leaf cell proliferation and expansion was made using a *cyc1At::GUS *reporter, but imaging was limited to histological sectioning and paradermal views [14]. Clearly, there exists a need for the visualisation and quantitative analysis of the cellular organisation of the intact leaf and all of its tissues in their three-dimensional context.

Currently, the most adequate and straightforward three-dimensional imaging methods for obtaining single-cell level resolution in plant biology are confocal and multiphoton laser scanning microscopy. Three-dimensional imaging of leaves has already been achieved by magnetic resonance imaging (MRI) [20-22], optical coherence microscopy (OCM) [23,24], high-resolution X-ray computed tomography (HRCT) [25] and optical projection tomography (OPT) [26]. However, none of these techniques provide the single-cell resolution required for the reconstruction and analysis of the cellular organisation of leaf tissues. They rather visualise the external morphology of the leaf at the organ level and thus allow the analysis of overall leaf volume and surface area. *In vivo *imaging using OCM does provide a tool for monitoring developmental changes at the organ level during leaf growth, while in OPT gene expression can be imaged, revealing for example the complete leaf venation pattern. MRI has been shown powerful in recording plant physiological processes such as water movement and the transport of assimilates and ions through vascular tissues. Particularly promising here is the recent development of MRI-positron emission tomography (PET) [27]. In contrast to these techniques, which require highly specialised equipment and handling, laser scanning microscopy is a very accessible imaging method, both in terms of equipment and usage. Confocal microscopes have been adopted at most research institutes and multiphoton systems become increasingly available via institute or regional wide imaging platforms.

It is generally accepted that multiphoton microscopy provides deeper depth penetration than confocal microscopy [28,29]. Plant leaves and various other plant organs are, however, notoriously difficult to image in depth because of weak penetration of light, which is due to an opaque cuticle and the presence of numerous light scattering molecules, mostly secondary metabolites, in the cuticle, cell wall and vacuoles [29]. Even with a multiphoton microscope, one cannot penetrate deeper than the epidermis and one layer of mesophyll cells [28] (authors' experience). In contrast, strong clearing of plant tissues allows optical sectioning down to 200 μm in depth using both techniques and is, in principle, only limited by the working distance of the objective [29,30]. This was recently shown by confocal imaging of Arabidopsis organs in gene expression analyses [31]. Image quality, certainly in depth, is nonetheless technique-dependent and multiphoton microscopy outperforms confocal imaging when it comes to signal-to-noise ratio, contrast and thus effective resolution [28]. Provided that optical sections are of high quality, *i.e*. high resolution and contrast, access is granted to volume-based quantitative data on leaf tissues and cell dimensions. Moreover, cells are shown within their three-dimensional context, which means that the structural interaction between cell types can be revealed.

In this paper, a procedure for the three-dimensional imaging and quantitative analysis of the structural properties of plant leaves is presented. It was developed and optimised for leaves of a wild-type accession of Arabidopsis (Col-4) grown in soil, and for a range of developmental stages, from initiation to senescence, representing differences in cell densities, cell dimensions and cuticle and cell wall properties. The resulting images covered the complete leaf thickness and were of high resolution and contrast throughout (when acquired by multiphoton laser scanning microscopy), allowing a detailed visualisation of the large diversity of leaf tissues. Here, the performance of the procedure was demonstrated for Arabidopsis leaf growth, focusing on four major structural and functional leaf tissues, the adaxial and abaxial epidermis and the palisade and spongy mesophyll. The procedure enabled the study of leaf expansion in surface area and thickness through specific tissue and cell variables such as the volumetric proportions of the epidermal and mesophyll tissues, cell density in these tissues, and cell dimensions, including volume, length and width.

## Results and Discussion

### Increased tissue permeability improves clearing and coloration of leaves

**Figure 1:**
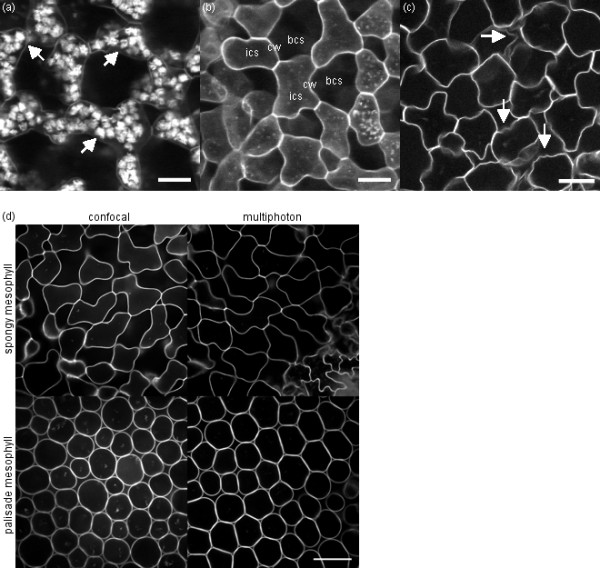
**Leaf image problems and image quality difference between confocal and multiphoton systems**. (a-c) Problems encountered upon three-dimensional imaging of leaves include: (a) starch granules (some indicated by arrows), (b) low contrast between cell walls (cw) and intracellular (ics) and intercellular spaces (bcs), and (c) wrinkling (some of it indicated by arrows) in Arabidopsis mesophyll cells (scale bars of 25 μm). (d) Single sections of an image stack of an Arabidopsis leaf (leaf 6, 16 days after initiation) acquired by confocal (left) or multiphoton (right) microscopy (scale bar of 50 μm for all images): sections in the spongy mesophyll at 35 μm in depth (top) and the palisade mesophyll at 125 μm in depth (bottom).

When working with leaves, the main hurdles to good quality images and cell measurements are the leaf cuticle and starch-containing plastids. The hydrophobic nature of the leaf cuticle renders any water-based treatment inefficient because of hampered penetration into the leaf. Better clearing and staining results are obtained at edges, where the integrity of the leaves is damaged and products can enter by diffusion. Starch often remains visible in cells as granules, even after classical clearing procedures, and hampers cell segmentation in subsequent image analysis steps (Fig. 1a). A protocol for the three-dimensional imaging of plant organs using confocal microscopy was described by Truernit *et al. *[31], but in the case of leaves, resulting images were of insufficient quality for cell segmentation and measurements, because of low signal-to-noise ratios and low contrast between cell walls and intra- and intercellular spaces (Fig. 1b). A hot ethanol treatment [31] for tissue clearing was not reliable for leaves of a range of developmental stages, even when prolonged, and important cell shrinkage or wrinkling phenomena in both the palisade and spongy mesophyll were observed (Fig. 1c). The main problem was most likely the leaf cuticle, which is fully-formed in soil-grown Arabidopsis (current experiments), but poorly developed and often discontinuous in *in vitro *plantlets (experiments by Truernit *et al. *[31]) [32]. The procedure described here aimed at providing efficient and consistent clearing and staining of leaves of different developmental stages, from initiation to maturity, and included a fixation and long-term conservation step to allow large-scale phenotyping experiments.

Whole leaves were fixed, conserved in 70% ethanol for up to 12 months and briefly rinsed in chloroform before clearing and coloration. Histochemical staining of the cuticle [33] confirmed the partial removal by these treatments of cuticular waxes, when leaves had been fixed in ethanol:acetic acid (3:1) or methanol:acetic acid:water (5:1:4). Aldehyde-based fixatives proved to be unsuitable because of continued and strong permeability issues and consequently difficulties in removing all of the chlorophyll and starch from plastids. Compared to fixation in methanol:acetic acid and freshly prepared ethanol:acetic acid, fixation in an aged solution of ethanol:acetic acid further improved tissue permeability to a crucial extent in the clearing and coloration steps of the procedure. This was observed as uniform staining over the complete leaf surface, instead of coloured patches or effective staining limited to damaged sample borders, an effective removal of starch granules and overall a significantly higher image quality. It was confirmed by gas chromatography that ethyl acetate had formed in the fixative, while the acid pH was maintained. Most likely, ethyl acetate aided in rendering the cuticle more permeable by a (partial) depolymerisation of the cutin polyester, a better removal of intracuticular waxes and a permeabilisation of cell walls. An alternative preparation method for the fixative was ethanol:acetic anhydride (3:1) which gave immediate formation of ethyl acetate.

The fastest and best clearing of leaf samples was obtained using sodium dodecyl sulphate and sodium hydroxide (SDS/NaOH), a classical cell lysis buffer, followed by digestion of residual starch by amylase. A modified periodic acid-pseudo-schiff treatment using propidium iodide as cell wall stain [29] proved to give reproducible results and sufficient fluorescent signal for high-contrast images of all leaf developmental stages. Chloral hydrate was applied in an extra clearing step after coloration. Although more time-consuming, clearing using only chloral hydrate could be envisaged for leaves with a low starch content or in case of GUS-stained leaves in gene expression studies. Finally, leaves were mounted in chloral hydrate or Hoyer's solution and imaged within one week. Samples could not be stored for longer in Hoyer's solution because of tissue compression upon drying and solidification, especially in the spongy mesophyll of mature leaves with large intercellular spaces.

### Multiphoton laser scanning microscopy outperforms confocal microscopy in image quality

Requirements for three-dimensional reconstruction and cell measurements were (i) coverage of the complete leaf thickness, from the adaxial to the abaxial epidermis, (ii) high lateral resolution or continuous cell walls and high contrast, and (iii) an acceptable axial resolution with, in particular, cells which are closed at their top and bottom, thus facilitating simple cell segmentation. Multiphoton microscopy is generally recommended over confocal microscopy for thick biological specimens [20]. In our experiments, leaf cells were fixed and rigorously cleared, which greatly improved tissue penetration and made that the complete leaf thickness, *i.e*. over 150 μm at the end of leaf blade expansion, could be imaged using confocal microscopy. The quality of the images was, however, compromised because of the high laser output required (from 4% for the first optical sections to 40% at >150 μm), which resulted in a lower contrast and a reduced signal-to-noise-ratio (Fig. 1d, confocal). Multiphoton microscopy, on the other hand, greatly improved image features, especially when used in the non-descanned mode of the system, where the fluorescent signal detection occurs externally (Fig. 1d, multiphoton). This means that the signal does not pass by the optical configuration of the scan head before detection and all emitted light, including scattered light, is detected. Typically, this mode is used for weak signal samples, but it has proven here to give images with superior contrast between cell walls and intra- and intercellular spaces, without line or frame averaging and without the need for significant laser output increases in depth.

The best axial resolution for our purposes, *i.e*. the three-dimensional reconstruction and quantitative analysis of the leaf epidermis and mesophyll, was obtained using a 40 × (NA 1.2) water-dipping objective lens with correction collar. It had a working distance of 280 μm (at cover slip thickness of 170 μm) which was sufficient for Arabidopsis leaf samples. Samples were placed on cover slips and then mounted on microscope slides in order to minimise the path length through the mounting medium on an inverted microscope. Spherical aberration, due to a refractive index mismatch between water as objective dipping medium and the chloral hydrate-glycerol-based mounting medium, was observed only when the density of trichomes was high thus creating a layer of mounting medium between the cover slip and sample.

The high image quality obtained by multiphoton microscopy meant that simple thresholding or automated cell segmentation could be used for the quantitative analysis of cell dimensions. Confocal images only allowed manual tracing and measurement of cells or required more complex segmentation algorithms. In the additional files, movies of multiphoton optical sections, going through a leaf from the adaxial to the abaxial epidermis or vice versa, are provided for young (Additional file 1) and near-mature Arabidopsis leaves (Additional file 2).

**Figure 2:**
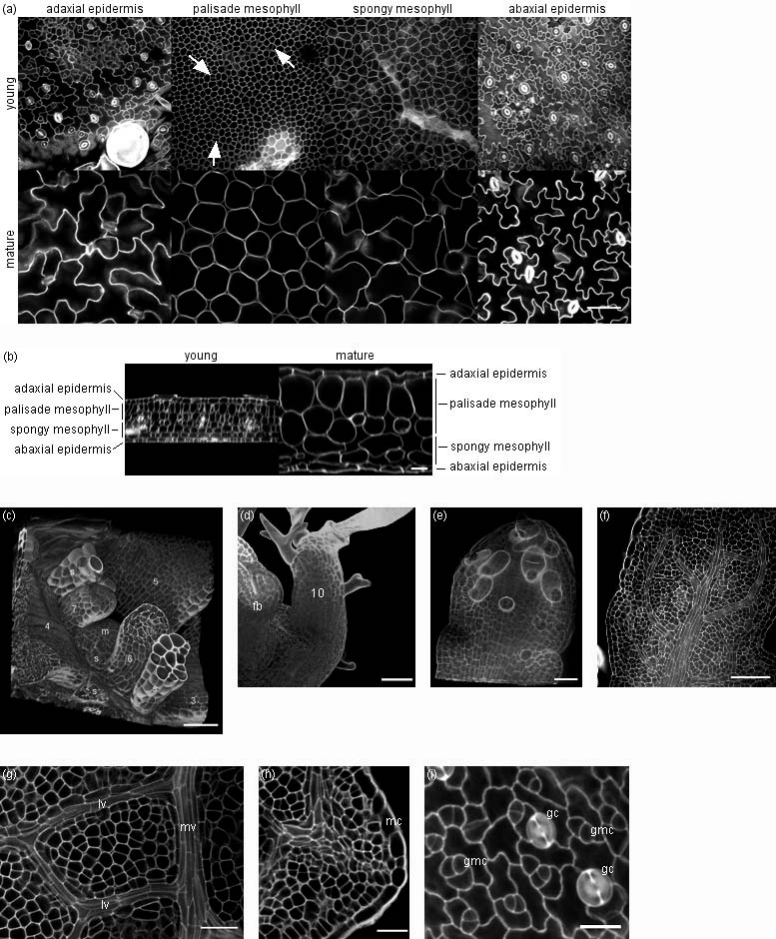
**Three-dimensional imaging of Arabidopsis leaves at different developmental stages using multiphoton microscopy**. (a) Single optical sections in, from left to right, the adaxial epidermis, the palisade mesophyll, the spongy mesophyll and the abaxial epidermis, taken from an image stack of a young leaf (leaf 6, 8 days after initiation, top row) and a mature leaf (leaf 6, 19 days after initiation, bottom row) (scale bar of 50 μm for all images at bottom right). Cell division planes are indicated by arrows. (b) Orthogonal views (xz) of the same image stacks: young leaf (left) and mature leaf (right) (scale bar of 25 μm for both images). (c-f) Three-dimensional imaging of leaves before emergence: (c) meristem (m) and leaves 3-7 with stipules (s) (12 days after sowing, scale bar of 20 μm), (d) leaf 10 and floral bud (fb) (18 days after sowing, scale bar of 20 μm), (e) leaf 9 (20 days after sowing, scale bar of 10 μm), (f) leaf 8 (20 days after sowing, scale bar of 50 μm). (g) Vein development in a young leaf (leaf 6, 11 days after initiation, mv-midvein, lv-lateral vein, scale bar of 25 μm). (h) Leaf serration in a young leaf (leaf 6, 7 days after initiation, mc-marginal cells, scale bar of 20 μm). (i) Guard cell development in a young leaf (leaf 6, 11 days after initiation, gc-guard cell, gmc-guard mother cell, scale bar of 15 μm).

In our experiments imaging of leaves was focused on the leaf blade and the structural organisation of the epidermal and mesophyll tissues. Figure 2 shows one optical section (xy) in each of these tissues (Fig. 2a) and an orthogonal view (xz) of the complete leaf thickness (Fig. 2b) of a young and a mature Arabidopsis leaf (leaf 6 of the rosette), 8 and 19 days after initiation, respectively (Additional file 3 shows the complete series between 8 and 22 days after initiation). The procedure described here allowed equally well the imaging of very young leaf stages (Fig. 2c-f) and of other particular aspects of leaf morphology, such as the development of vein structure (Fig. 2g), serrations and marginal cells (Fig. 2h), and guard cell initiation and development (Fig. 2i).

Whether the procedure could also be used for other species was tested on leaves of apple (*Malus domestica *Borkh.) which have higher epidermal and mesophyll cell densities and larger intercellular spaces in the spongy mesophyll compared to Arabidopsis. Sample preparation and imaging was successful and the difference between Arabidopsis and apple leaf samples lay primarily in the duration of the main clearing step (SDS/NaOH, 6 h), not in the number of treatments required. In the additional files, movies of multiphoton optical sections, going through a leaf from the adaxial to the abaxial epidermis or vice versa, are provided for young (Additional file 4) and mature apple leaves (Additional file 5). Additional file 6 shows single optical sections in the adaxial epidermis, palisade and spongy mesophyll, and abaxial epidermis of young and mature apple leaves and an orthogonal view (xz) of the complete thickness of these leaves.

### Three-dimensional visualisation and image analysis for the extraction of tissue and cell dimensions

**Figure 3:**
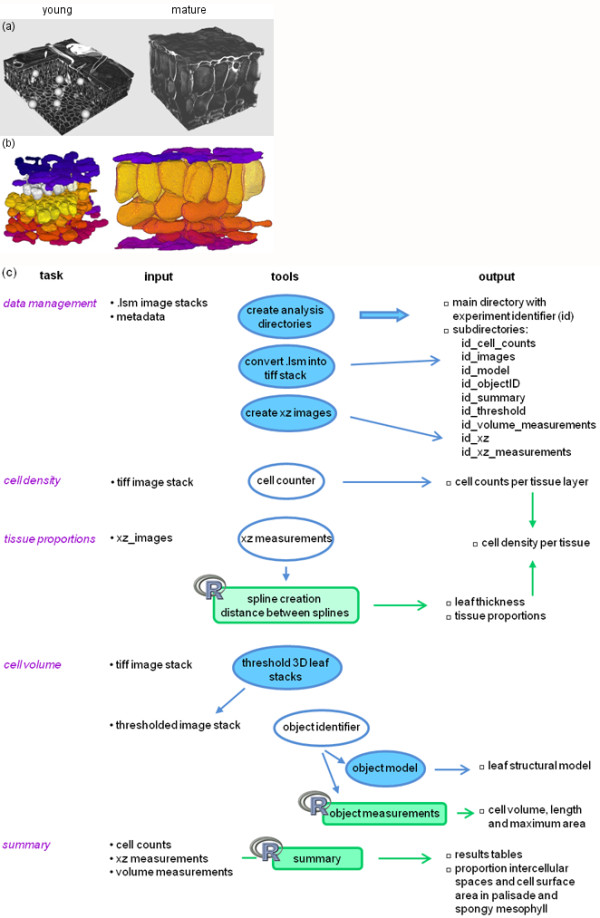
**Three-dimensional visualisation and image analysis**. (a) Three-dimensional visualisation in the MedINRIA ImageViewer software of a young (leaf 6, 8 days after initiation, left) and a mature Arabidopsis leaf (leaf 6, 19 days after initiation, right). (b) Three-dimensional structural model of a young (leaf 6, 10 days after initiation, left) and a mature Arabidopsis leaf (leaf 6, 22 days after initiation, right). (c) Image analysis flow diagram: ImageJ macros are indicated in blue (blue background-fully automated, white background-semi-automated) and R scripts in green.

For the visualisation of image stacks in three dimensions we opted for the open-source ImageJ 3D viewer plugin [34] and MedINRIA's ImageViewer module [35] (Fig. 2c-e and Fig. 3a, respectively). Orthogonal views (xz) were obtained using the ImageJ Ortview plugin [34] (Fig. 2b).

The main leaf growth variables that we were interested in, in view of the quantitative analysis of leaf expansion at the organ, tissue and cell level, were leaf thickness, the volumetric proportions of epidermal and mesophyll tissues, cell density in these tissues, cell dimensions (including volume, length and area or width), and finally the proportion and volume of intercellular spaces and cell surface area of mesophyll tissue. To accommodate for large sample numbers and image stacks of 90-290 Mb, ImageJ macros were developed for the creation of an ordered data structure and a standardised measurement procedure, and for the automation of image analysis tasks. The flow diagram of the image data management and analysis procedure consisted of four major tasks (Fig. 3c): (i) creation of data storage files for the raw images, treated images and all measurements performed, (ii) cell counting for the calculation of cell density, (iii) delineation of tissues in orthogonal (xz) views for leaf thickness measurement and the calculation of tissue proportions, and (iv) cell segmentation, including thresholding using k-means clustering and semi-automated cell identification, for the creation of a three-dimensional structural model and the measurement of cell dimensions. Examples of leaf structural models are shown in Fig. 3b. The proportion of intercellular spaces in the palisade and spongy mesophyll was estimated based on the volumetric proportion of the tissue, cell density and cell volume. Calculations and data analyses were performed in the statistical computation system R [36] by means of scripts, which further allowed for standardisation. Compared to images of other types of biological specimens, stacks of leaf optical sections were large, cells were numerous and irregular in shape, and intercellular spaces were big, all of which compromised automated cell segmentation.

### Multiphoton imaging for the quantitative analysis of growth variables at the organ, tissue and cell level

#### Organ level growth variables corresponded well between different techniques

Leaf expansion was analysed for leaf 6 of the Arabidopsis rosette using in parallel two-dimensional histological sectioning and the three-dimensional imaging procedure described here. Up to 40 three-dimensional image stacks were acquired per day which demanded a sample preparation time of 5 h spread over 3 days. An equal number of two-dimensional images required a preparation time of 14 h spread over 8 days. Also, in case of three-dimensional imaging, whole leaves or small seedlings were fixed, stained and mounted, which gave a higher flexibility in the choice of imaging positions within the leaf or the seedling. Moreover, this was decided on at the time of image acquisition, not at sample collection or during sectioning.

**Figure 4:**
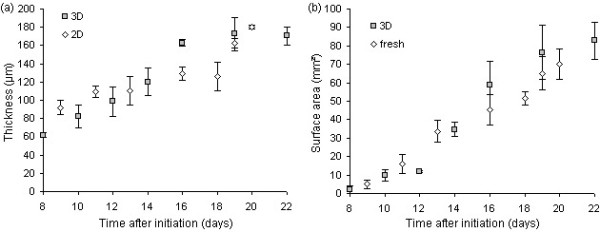
**Thickness and surface area of expanding leaves measured by different techniques**. (a) Thickness (average ± standard deviation) measured in the middle of leaf 6 of the Arabidopsis rosette on transversal views obtained by histological sectioning (2D) or three-dimensional imaging (3D). (b) Surface area (average ± standard deviation) of leaf 6 of the Arabidopsis rosette measured on scans of fresh leaves (fresh) or after three-dimensional imaging (3D).

A high degree of correspondence between leaf thickness measurements on histological sections and three-dimensional images was obtained (Fig. 4a). Thickness measured within a three-dimensional image stack varied by <1%, while 2% variation was noted between image stacks within a certain region of the leaf, such as the base, middle or tip.

**Figure 5:**
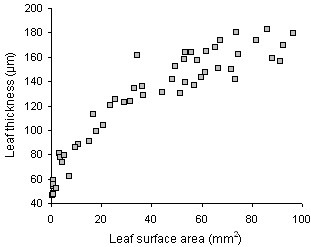
**Leaf expansion in surface area and thickness**. Scatter plot of thickness versus surface area of individual leaves (leaf 6 of the Arabidopsis rosette) between 8 and 22 days after leaf initiation.

Leaf surface area measured on scans of leaf samples on microscope glasses corresponded equally well with measurements on scans of fresh leaf samples collected during the course of the experiment (Fig. 4b), suggesting the absence of morphological modifications by the chemical treatments. Currently, the assessment of leaf phenotypes is primarily based on (expansion in) surface area. A study of the correlation between surface area and any other variable measured in leaves may reveal whether surface area alone can adequately describe the phenotype. For example, the plot of thickness versus surface area of expanding leaves has revealed the absence of a linear correlation between both variables, demonstrating that leaf expansion in surface area and thickness occurs asynchronously. Moreover, larger leaves are not necessarily thicker (Fig. 5).

#### Tissue level growth variables were reliably measured for all layers of mesophyll tissue

**Figure 6:**
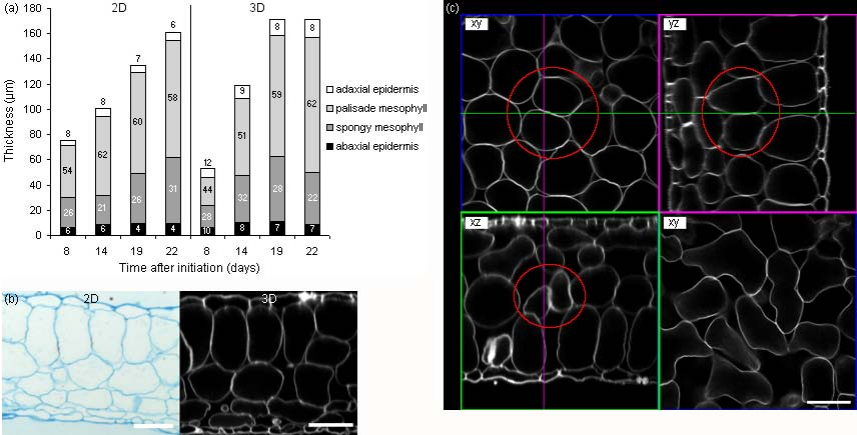
**Leaf tissue expansion measured on two- and three-dimensional images**. (a) Thickness and proportions of leaf thickness (average %, indicated inside bars) occupied by, from top to bottom, the adaxial epidermis, the palisade mesophyll, the spongy mesophyll and the abaxial epidermis, in the middle of leaf 6 of the Arabidopsis rosette as measured by histological sectioning (2D, left) and three-dimensional imaging (3D, right). (b) Transversal (xz) views of leaf 6 of the Arabidopsis rosette (19 days after initiation) obtained by histological sectioning (2D, left) and three-dimensional imaging (3D, right, scale bar of 50 μm for both images). (c) Paradermal (xy) and transversal (xz, yz) views of a leaf image stack illustrating the difficulty in the assessment of palisade and spongy mesophyll cell identity using transversal views only. The palisade mesophyll cells encircled in red could easily be mistaken for spongy mesophyll cells based on the xz-view only (bottom left). Their identity is clear in the xy-(top left) and yz-views (top right). True spongy mesophyll cells are shown for a comparison of cell shapes (xy, bottom right, scale bar of 50 μm).

The proportions of epidermal and mesophyll tissues in expanding leaves as measured in thickness (histological sectioning) or volume (three-dimensional imaging) did not correspond well between both techniques, especially for mesophyll tissue (Fig. 6a). This was due to the difficulty of assessing cell characteristics (palisade or spongy) in transversal views (xz) only (Fig. 6b). In three-dimensional image stacks, the extent of a certain tissue and the identity of each individual cell was determined using both transversal (xz) and paradermal (xy) views. Based on cell shape in the paradermal view, we found two layers of palisade mesophyll cells (round) versus two loosely arranged layers of spongy mesophyll cells (lobed) in our growing conditions. In transversal views cells of the second layer of palisade mesophyll could easily be confused for spongy mesophyll, depending on the position in the cell (Fig. 6c).

**Figure 7:**
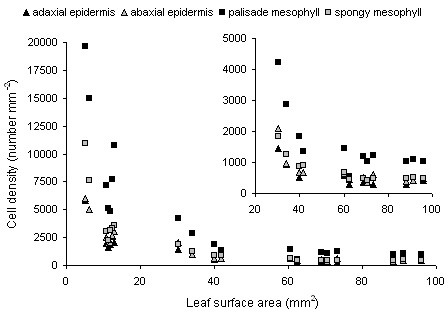
**Cell density determined for both epidermal and mesophyll tissues**. Scatter plot of cell density versus leaf surface area of individual leaves (leaf 6 of the Arabidopsis rosette, between 8 and 22 days after leaf initiation) as determined for the adaxial and abaxial epidermis, and the palisade and spongy mesophyll. The inset is a zoom on the dataset and shows cell densities for leaf surface areas between 20 and 100 mm^2^.

Cell density is a variable used in the comparison of leaf phenotypes in genotype × environment interactions and in models of leaf size control [10,11], but is generally limited to epidermal cells and determined using brightfield or differential interference contrast microscopy. Three-dimensional imaging has given access to cell density data on internal tissues as well, which is important in the assessment of plant and organ signal integration at the cellular level and in studies on cell-cell communication in epidermal and mesophyll tissues [37,38]. Figure 7 shows the scatter plot of the cell densities in the adaxial and abaxial epidermis and the palisade and spongy mesophyll versus surface area of the expanding leaf 6 of the Arabidopsis rosette. The highest cell densities were observed in the palisade mesophyll. The rapid decrease in cell densities (leaves of 5-40 mm^2^) corresponded to the phase of high relative leaf expansion rates, and was followed by a phase of slowly decreasing cell densities before final leaf surface area was reached (Fig. 7, inset).

#### Cell level growth variables revealed a large range of cell sizes in the mesophyll

**Figure 8:**
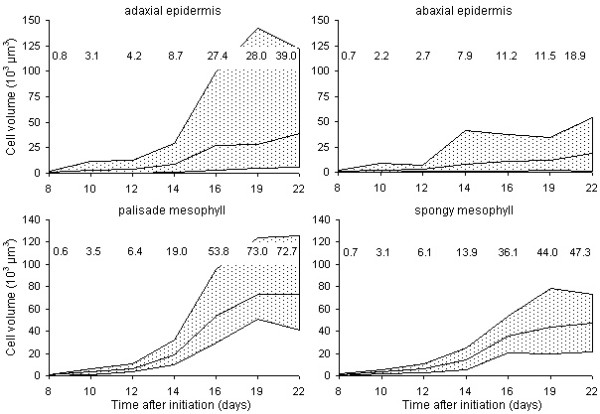
**Cell volumes measured in both epidermal and mesophyll tissues**. Range of cell volumes observed during expansion in the adaxial epidermis (top left), the abaxial epidermis (top right), the palisade mesophyll (bottom left) and the spongy mesophyll (bottom right) of leaf 6 of the Arabidopsis rosette. The graphs represent the minimum and maximum cell volume measured; the lines in the middle of the graphs indicate the average cell volume. Its value is shown for the different time points (days after initiation of leaf 6).

Three-dimensional imaging provided access to volumetric cell dimensions for individual cells of all layers of mesophyll tissue via the construction of a leaf structural model. The volume of intercellular spaces and the cell surface area per volume of mesophyll tissue were also estimated and represent, together with mesophyll cell volumes, important parameters in leaf biochemical and physiological studies, which in general are based on physical measurements only or limited to leaf fresh and dry weight [39,40]. The overall observation at the cell level was the large range of cell volumes in epidermal as well as mesophyll tissues, which increased substantially during leaf expansion, especially in the adaxial epidermis and the palisade mesophyll (Fig. 8).

**Figure 9:**
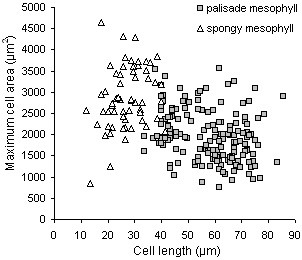
**Length and area of mesophyll cells in fully expanded leaves**. Scatter plot of cell length versus maximum cell area of individual cells in the palisade and spongy mesophyll of leaf 6 of the Arabidopsis rosette at the end of leaf surface area expansion (19-22 days after initiation).

For each individual cell measured in volume, additional parameters such as length, maximum area or width, and cell surface area were determined. The scatter plot of maximum area versus length of individual palisade and spongy mesophyll cells is a further demonstration of the diversity of cell dimensions detected in fully expanded leaves (Fig. 9).

**Figure 10:**
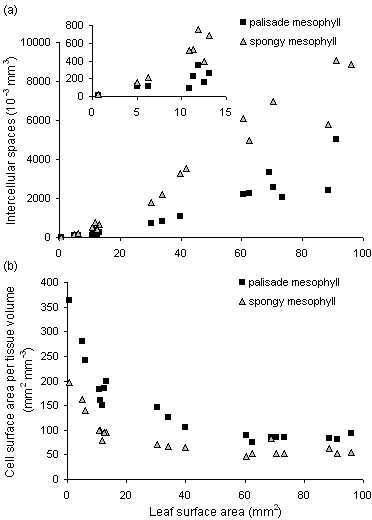
**Intercellular spaces and cell surface area in the palisade and spongy mesophyll**. Scatter plot of the volume of intercellular spaces (a) and cell surface area per tissue volume (b) versus leaf surface area in the palisade and spongy mesophyll of individual leaves (leaf 6 of the Arabidopsis rosette, between 8 and 22 days after leaf initiation). The inset in (a) is a zoom on the dataset and shows the volume of intercellular spaces for leaf surface areas between 0.5 and 15 mm^2^.

Tissue proportions, cell densities and cell volumes allowed the estimation of the proportion and volume of intercellular spaces in the palisade and spongy mesophyll and the total cell surface area per tissue volume. As expected, the spongy mesophyll contained the largest volume of intercellular spaces from the onset of leaf expansion (Fig. 10a). Mesophyll cell surface area per tissue volume decreased during expansion, synchronously to cell density, and reached near the end of leaf expansion values of 85 ± 6 and 57 ± 11 mm^2 ^mm^-3 ^for the palisade and spongy mesophyll, respectively (Fig. 10b). Mesophyll cell surface area in contact with intercellular spaces is a further specification of this variable frequently used in physiological studies [41]. The automated calculation of this variable, based on three-dimensional cells and thus without the incorporation of assumptions on cell dimensions, will be feasible through basic image analysis once automated cell segmentation has been achieved.

## Conclusions

In the procedure presented here, the critical steps in the preparation of leaf samples for three-dimensional imaging proved to be tissue permeabilisation during fixation (to obtain better clearing and staining), and the removal of cellular contents using SDS/NaOH and amylase. Both confocal and multiphoton microscopy enabled imaging of the entire leaf thickness, but the latter provided greater image quality and thus access to quantitative data on tissue and cell dimensions. The image analysis tools required to obtain these data were developed. It was demonstrated that through three-dimensional imaging and image analysis, parameters on internal tissues can be included in studies of leaf expansion and the characterisation of leaf phenotypes, which until now were based mainly on leaf surface area and epidermal cell density and area.

This paper focused on a particular set of leaf tissues, but the imaging technique is not limited here. Other tissues can equally be visualised and samples may include leaves in early stages of development (cell proliferation) or near senescence. It has also been shown that the sample preparation procedure is effective on leaves of other species.

Three-dimensional imaging will further be applied in the determination of the minimum set of growth variables required for the adequate assessment of leaf phenotypes, which can then be incorporated in models of leaf size control. In general, the quantitative analysis of epidermal and mesophyll tissue and cell properties, during development or at a specific stage, could provide the data required for a more comprehensive interpretation of genetic and environment-induced modifications, biochemical analyses and "-omics" results, and for the initialisation of leaf development and functioning models. Finally, three-dimensional visualisation of the leaf's cellular organisation may reveal tissue and cell interactions not observed previously.

## Methods

### Plant material and growth conditions

Arabidopsis Col-4 (N933, http://Arabidopsis.org.uk was grown in a mixture (1:1) of a loamy soil and organic compost in the PHENOPSIS phenotyping platform [42,43] under controlled environmental conditions: air temperature of 21°C, 8 h photoperiod, incident light intensity of 230 μmol m^2 ^s^-1 ^provided by a bank of cool-white fluorescent tubes and HQI lamps, 70% air humidity and 40% soil humidity.

### Arabidopsis leaf growth assessment

Leaf 6 samples were collected every 2-3 days from leaf initiation until the end of expansion. Leaf 6 surface area was determined for five rosettes per time point, first under a stereomicroscope (Leica Wild F8Z, http://www.leica-microsystems.com at magnification 160 × using dedicated image analysis software (Bioscan-Optimas V 4.10; Edmonds, WA, USA) and afterwards on scans of leaves of dissected rosettes using an ImageJ macro [43]. Two times six leaf samples per time point were harvested for the measurement of leaf thickness and other growth variables by histological sectioning and three-dimensional imaging.

### Fixation, clearing and staining procedure for three-dimensional imaging

Whole seedlings or leaves were fixed in 5-8 ml of an aged solution of ethanol:acetic acid (3:1) (stored for a minimum of 4 months at 4°C) or a solution of ethanol:acetic anhydride (3:1) with a drop of Tween-20 (50 μl). They were put under vacuum for 1 h and left on a shaker at 4°C for 48 h. Afterwards, they were rinsed in 50% and 70% ethanol. Leaf samples were conserved in 70% ethanol at 4°C. The procedure for clearing and staining was independent of leaf age or rank and included the following steps: (i) day 1, 10 min chloroform treatment, rinse in 70% ethanol and progressive rehydration for a transition to water-based treatments, 15 min clearing in SDS/NaOH, rinse in water and overnight amylase treatment at 37°C; (ii) day 2, rinse in water, 40 min periodic acid treatment, rinse in water and 6 h of staining in pseudo-schiff-propidium iodide followed by an overnight rinse in water; (iii) day 3, clearing in chloral hydrate (min 4 h) and montage in Hoyer's solution, if required. The products and concentrations were: absolute chloroform, 1% SDS and 200 mM NaOH, 20 mM PBS pH 7.0, 2 mM NaCl and 0.25 mM CaCl_2_, 0.01% amylase in PBS (SIGMA A4551, http://www.sigmaaldrich.com, 1% periodic acid, freshly prepared pseudo-schiff consisting of 100 mM Na_2_S_2_O_5 _and 0.15 N HCl, 0.01% propidium iodide added to the pseudo-schiff solution at the time of staining, saturated chloral hydrate solution (200 g chloral hydrate, 20 ml glycerol and 30 ml water), Hoyer's solution (10 g arabic gum, 40 ml MilliQ water, 10 ml glycerol, 100 g chloral hydrate). Leaves were positioned on cover slips and then turned and mounted on microscope slides. This was done to limit the distance between the sample and cover slip, thereby optimising for the limitation imposed by the working distance of the objective and the path of the laser and emitted light.

### Microscope and imaging conditions

Samples were imaged on a Zeiss Axiovert 200 M LSM Meta 510 NLO http://www.zeiss.com/micro equipped with a Coherent Ti:Sa laser Chameleon Ultra II http://www.coherent.com at the Montpellier RIO imaging platform http://www.mri.cnrs.fr. Propidium iodide was excited with the 488 nm Ar laser line of the confocal system or 790 nm of the chameleon laser of the multiphoton system. Parameters for confocal acquisition were: HFT KP 700/488 multiple beam splitter, NFT 545 dichroic beam splitter, LP560. Parameters for multiphoton acquisition were: HFT KP 650 main dichroic beam splitter, non-descanned detection KP 685 secondary dichroic beam splitter, FT 560 filter wheel, BP575-640. A C-Apochromat 40 ×/1.2 W Corr objective lens was routinely used. Scans were performed at 1024 × 1024 pixels, 8-bit, using bi-directional scanning and a pixel-time of 3.2 μs. No line or frame averaging was applied. Scans were performed at 0.8 μm intervals in depth, which gave a voxel size of 0.22*0.22*0.8 μm (xyz). Imaging of the entire thickness of a leaf, 50 to 200 μm, took 4 to 17 min. Image stacks were produced routinely for the leaf base, middle or tip along the longitudinal axis, and approximately midway between the leaf midvein and margin.

### Visualisation and image analysis

Visualisation of image stacks was done in ImageJ [34] or the MedINRIA's ImageViewer module [35]. For the latter, tiff format images were converted into the NIfTI format by means of a bat file performing the conversion using a dedicated program (P. Fillard, INRIA, Sophia-Antipolis, France). For the quantitative analysis of tissue and cell dimensions in image stacks, specifically developed ImageJ macros and R scripts [36] were used. These are available from the corresponding author on request.

### Histological sectioning

Leaves were cut into small pieces, fixed in 1% glutaraldehyde, 2% paraformaldehyde, 1% caffeine in 0.1 M PBS pH 7.0 for 24 h at 4°C, rinsed twice in 70% ethanol and conserved in 70% ethanol at 4°C. Because of their small size, leaf pieces were mounted in 1.7% agar before dehydration in ethanol and inclusion in resin. Transverse sections of 3.5 μm were cut on a Leica microtome RM2255 http://www.leica-microsystems.com and stained in 1% alcian blue 8GX in a sodium citrate/HCl pH 3.5 buffer. They were documented using a 20 × or 10 × objective lens on a Leica DM 4500 brightfield microscope http://www.leica-microsystems.com equipped with a Hamamatsu camera http://www.hamamatsu.com and Volocity imaging software http://www.cellularimaging.com. For each leaf piece, taken at approximately 50% between the leaf base and tip along the longitudinal axis, a central zone between the leaf midvein and leaf margin was photographed. Thickness was measured using a dedicated ImageJ macro (M. Lartaud, CIRAD, Montpellier, France).

## Competing interests

The authors declare that they have no competing interests.

## Authors' contributions

NW developed the sample preparation procedure in collaboration with JCP, optimised and performed the sample imaging, designed the image analysis procedure, carried out the image analysis and interpretation of data, and drafted the manuscript. GC and JLV provided support on sample preparation and imaging and supervised histological sectioning. CG conceived of the study, participated in its design and coordination and helped to draft the manuscript. CM designed and carried out the biological experiments, contributed in the interpretation of data and helped to draft the manuscript. All authors have read and approved the final manuscript.

## Supplementary Material

Additional file 1 **Image stack of an Arabidopsis leaf early in leaf blade expansion**. Optical sectioning of an Arabidopsis Col-4 leaf 6 at 9 days after initiation (16 h photoperiod) using multiphoton microscopy. Click here for file

Additional file 2 **Image stack of an Arabidopsis leaf near the end of leaf blade expansion**. Optical sectioning of an Arabidopsis Col-4 leaf 6 at 16 days after initiation (16 h photoperiod) using multiphoton microscopy. Click here for file

Additional file 3 **Three-dimensional imaging of Arabidopsis leaves using multiphoton microscopy**. Single optical sections in, from top to bottom, the adaxial epidermis, the palisade mesophyll, the spongy mesophyll and the abaxial epidermis, taken from image stacks of leaf 6 of the Arabidopsis rosette at 8-22 days after initiation (dai). Click here for file

Additional file 4 **Image stack of an apple leaf early in leaf blade expansion**. Optical sectioning of the third leaf from the top of an apple graft (Granny Smith) using multiphoton microscopy. Click here for file

Additional file 5 **Image stack of a fully expanded apple leaf**. Optical sectioning of the ninth leaf from the top of an apple graft (Granny Smith) using multiphoton microscopy. Click here for file

Additional file 6 **Three-dimensional imaging of apple leaves using multiphoton microscopy**. (a) Single optical sections in, from left to right, the adaxial epidermis, the palisade mesophyll, the spongy mesophyll and the abaxial epidermis, taken from an image stack of a young (the first unfolded leaf on the axis of a Starkrimson graft, top row) and mature apple leaf (the ninth leaf from the top of the same axis, bottom row) (scale bar of 50 μm for all images). (b) Orthogonal views (xz) of the same image stacks: young (left) and mature (right) apple leaf (scale bar of 25 μm for both images). Click here for file
