# Supplementary figures and images for: High-contrast three-dimensional imaging of the Arabidopsis leaf enables the analysis of cell dimensions in the epidermis and mesophyll
Source: Plant Methods. 2010 Jul 2;6:17. doi: 10.1186/1746-4811-6-17 (PMC2909956; doi:10.1186/1746-4811-6-17)

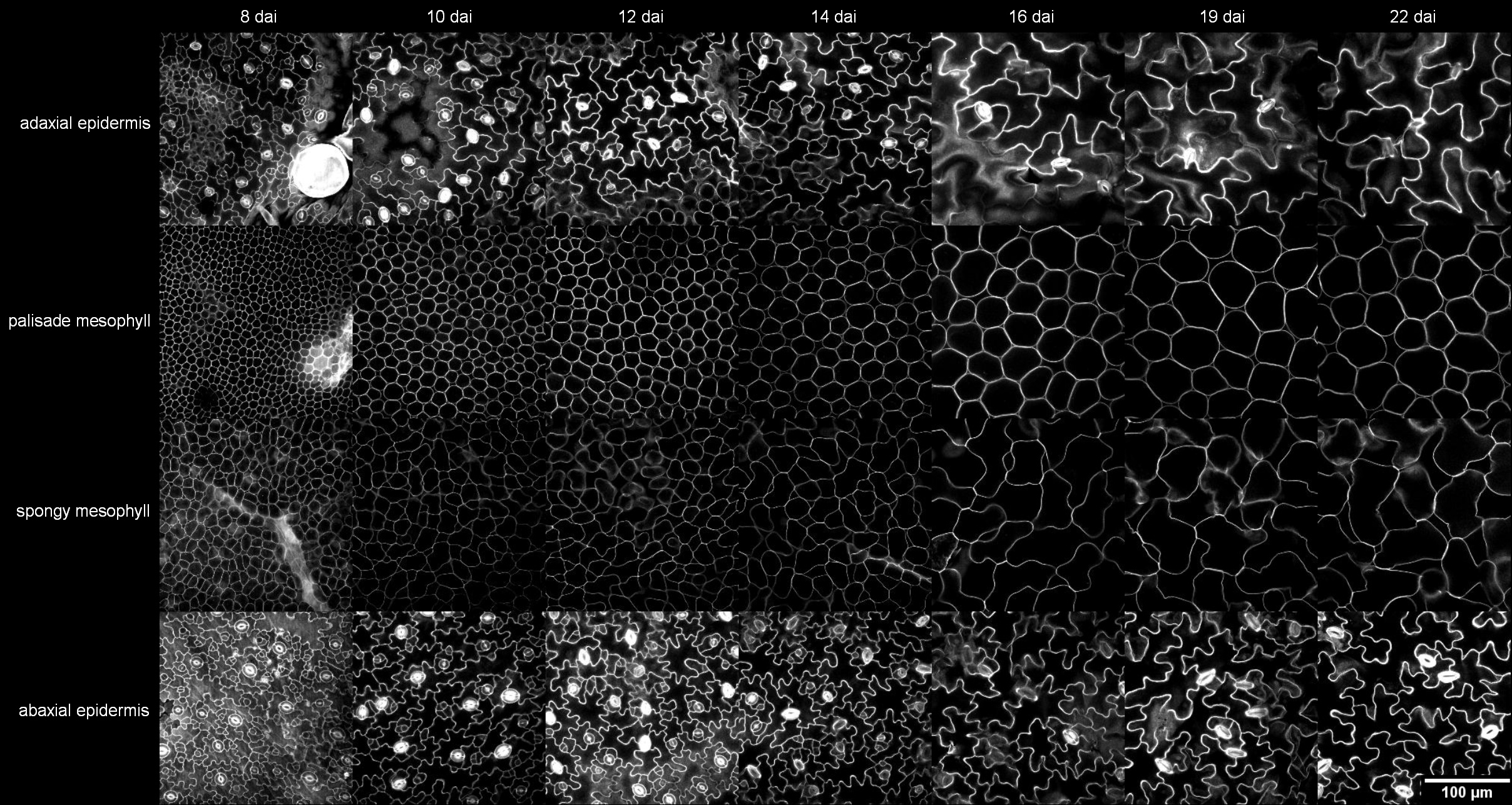

Supplement: Additional file 3 — Three-dimensional imaging of Arabidopsis leaves using multiphoton microscopy. Single optical sections in, from top to bottom, the adaxial epidermis, the palisade mesophyll, the spongy mesophyll and the abaxial epidermis, taken from image stacks of leaf 6 of the Arabidopsis rosette at 8-22 days after initiation (dai). [file 1746-4811-6-17-S3.TIFF]

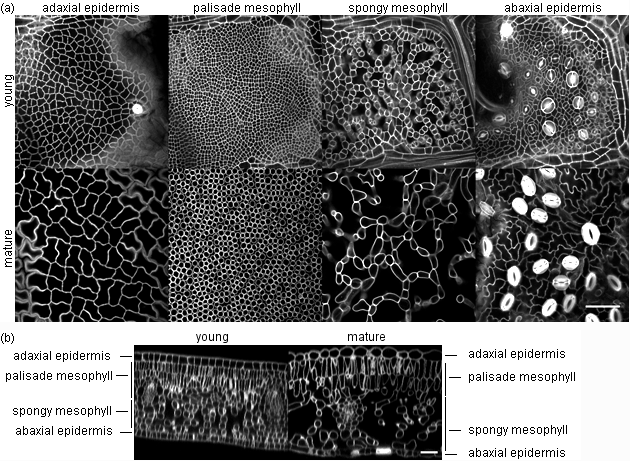

Supplement: Additional file 6 — Three-dimensional imaging of apple leaves using multiphoton microscopy. (a) Single optical sections in, from left to right, the adaxial epidermis, the palisade mesophyll, the spongy mesophyll and the abaxial epidermis, taken from an image stack of a young (the first unfolded leaf on the axis of a Starkrimson graft, top row) and mature apple leaf (the ninth leaf from the top of the same axis, bottom row) (scale bar of 50 μm for all images). (b) Orthogonal views (xz) of the same image stacks: young (left) and mature (right) apple leaf (scale bar of 25 μm for both images). [file 1746-4811-6-17-S6.TIFF]
